# Influence of endodontic treatment quality on apical periodontitis in maxillary and mandibular molars: A retrospective CBCT study

**DOI:** 10.6026/973206300214720

**Published:** 2025-12-15

**Authors:** Priyatam Karade, Sangeeta Nawal, Parul Mehta, Prateek Singh Kheriya, Kavita Pankaj sonwane, Deepika Yadav

**Affiliations:** 1 Department of conservative dentistry and endodontics, Bharati Vidyapeeth Dental College and Hospital, Sangli, Maharashtra, India; 2 Department of Conservative dentistry and Endodontics, Dr Nawals clinic 163 Vivek vihar JLN Road, Jaipur, Rajasthan, India; 3 Department of Conservative Dentistry, Eklavya Dental College, Kotputli, Jaipur, Rajasthan, India; 4 Department of Conservative Department of Dentistry and Endodontics, Modern Dental College and Research Centre, Indore, Madhya Pradesh, India; 5 Department of conservative Dentistry & Endodontics, D Y Patil Dental school, Lohegaon, Pune, Maharashtra, India; 6 Department of Conservative Dentistry and Endodontics, Institute of Dental Sciences, SOA University, Bhubaneshwar, Odisha, India

**Keywords:** Apical periodontitis, Cone-beam computed tomography (CBCT), endodontic treatment quality, root canal obturation, missed canals

## Abstract

The effect of endodontic treatment quality on apical periodontitis (AP) in maxillary and mandibular molars was evaluated in this retrospective CBCT study. AP was found in 54% of the 174 treated molars that were evaluated; it was more common in mandibular molars. Significantly lower AP prevalence was linked to adequate obturation length and density. Inadequate coronal restorations and missed canals were strongly associated with increased AP incidence. Thus, data shows the importance of proper coronal sealing and high-quality root canal therapies are for long-term success.

## Background:

Because it can detect apical periodontitis more accurately than traditional periapical and panoramic radiography, cone-beam computed tomography (CBCT) has become a valuable diagnostic tool in endodontics [1]. Research indicates that periapical health is significantly influenced by the length of root canal obturation, and CBCT is more accurate in identifying lesions associated with inadequate fillings [2]. The prudent and evidence-based use of CBCT in endodontics, especially when traditional imaging is insufficient, has been emphasized by the European Society of Endodontology [3]. Periapical healing is closely related to the technical quality of root canal therapy, and CBCT has demonstrated superiority in evaluating obturation quality and periapical status [4]. Studies show that inadequate coronal sealing and poorly condensed fillings substantially increase the prevalence of apical periodontitis [5]. Furthermore, CBCT enhances the detection of untreated or missed canals, which are major etiological factors in persistent periapical pathology [6]. Technical errors such as missed canals, underfilling, and overfilling remain frequent and are strongly associated with apical lesions [7]. Short or overextended obturations have been consistently linked to higher rates of periapical radiolucencies, reinforcing the critical relationship between obturation length and apical periodontitis [8]. Missed canals, in particular, continue to be among the strongest indicators of persistent infection [9]. Large population-based CBCT studies provide robust epidemiological evidence showing that the quality of root canal fillings significantly influences the prevalence of apical radiolucencies [10]. Moreover, recent cross-sectional analyses confirm that missed canals remain common and are closely associated with periapical lesions, emphasizing the clinical importance of anatomical knowledge and the selective use of CBCT for treatment evaluation [11]. Therefore, it is of interest to study of endodontic treatment quality on apical periodontitis in maxillary and mandibular molars.

## Methodology:

Cone-beam computed tomography (CBCT) scans obtained from patients treated at the Department of Oral and Maxillofacial Radiology and Endodontics between 12 months were examined in this retrospective cross-sectional study. All scans were performed for purposes other than this study, such as diagnosis or treatment planning. Using two-proportion analysis, a sample size of 82 teeth per group (164 total) was determined in order to detect a clinically significant difference in the prevalence of apical periodontitis (AP) between groups based on endodontic treatment quality (20% prevalence in adequate vs. 40% in inadequate cases, α = 0.05, power = 80%). For attrition and subgroup analyses, the sample size was slightly expanded. Included were scans showing complete mandibular and maxillary first and second molars with radiopaque root filling material. Patients who had diagnostic-quality scans free of significant artefacts and were at least 18 years old were eligible. Root resections, hemisections, retreatments, implants that altered anatomy, recent endodontic therapy (less than six months), vertical root fractures, and advanced periodontal bone loss were all considered exclusion criteria. Balanced representation was guaranteed through random stratified sampling by jaw (maxilla/mandible) and tooth (first/second molar). The CBCT acquisition parameters (model, voxel size, field of view, exposure, and reconstruction software) were noted after patient identifiers were eliminated. The main result was the presence of AP, which is a radiolucent region that is adjacent to the apex and measures ≥0.5 mm. volumetric lesion size and severity (CBCT-PAI) were secondary outcomes. Obturation length (adequate 0-2 mm short, underfilled >2 mm, overfilled beyond apex), density (continuous, void-free filling vs. inadequate), missed canals (particularly MB2 in maxillary molars), and coronal restoration integrity were used to evaluate the quality of the treatment. If every criterion was met, the overall quality was good; if not, it was poor. Axial, sagittal, coronal, and three-dimensional reconstructions of every tooth were assessed. Scans were evaluated independently by two calibrated examiners (Cohen's kappa ≥0.8); disagreements were settled by consensus or by a third examiner. Segmentation and manual outlining were used to measure the lesion volume and intraclass correlation was used to verify intra- and inter-examiner reliability. Tooth number, jaw, CBCT unit details, patient demographics, treatment quality, AP presence, severity, and volume, as well as other findings (e.g., perforations, separated instruments) were among the data gathered. Descriptive statistics, chi-square/Fisher's exact tests, t-tests/Mann-Whitney U, and logistic regression were used in the analyses (SPSS/Stata/R) to look for relationships between treatment quality and AP (adjusted ORs, 95% CI). Differences in voxel size and jaw/tooth type were examined using stratified and sensitivity analyses. By using pre-existing scans that were anonymised and radiation-free, ethical standards were upheld. The study complied with STROBE regulations. Retrospective design, CBCT parameter variability, and a limited treatment history are among the limitations. Nevertheless, the design allows for a thorough evaluation of the quality of endodontic treatment and how it affects AP in molars.

## Results:

162 of the 186 CBCT scans that were screened during the study period met the inclusion requirements. 174 root canal-treated molars, including 92 maxillary and 82 mandibular molars, were chosen for assessment. Patients in the study sample range in age from 19 to 68 years, with a mean age of 41.7 ± 12.6 years. The distribution of sexes was almost equal, with 51.9% of patients being female and 48.1% being male. There was no discernible gender difference in the prevalence of AP (p > 0.05). A total of 54.0% of the 174 teeth had apical periodontitis (AP), which was found in 94 of them. The prevalence of AP was marginally higher in mandibular molars (59.8%) than in maxillary molars (48.9%), although this difference was close to but fell short of statistical significance (p = 0.08) (Table 1 - see PDF). First molars had a slightly higher proportion of AP (56.3%) when stratified by tooth type than second molars (50.9%), which may indicate that first molars are more vulnerable to apical pathology after root canal therapy. The evaluation of the quality of endodontic treatment showed that 72 teeth (41.4%) had underfilling (>2 mm short of the apex) or overfilling beyond the apex, whereas 102 teeth (58.6%) had an adequate obturation length. While the remaining 78 teeth (44.8%) showed either insufficient compaction or the presence of voids, 96 teeth (55.2%) had adequate obturation density, which is defined as homogenous fillings without visible voids. The prevalence of AP was significantly lower in teeth with both adequate obturation length and density (32.6%) than in teeth with inadequate treatment (71.1%), and the difference was highly significant (p < 0.001) (Table 2 - see PDF). In addition, 42 molars (24.1%) had missing canals, with maxillary molars (especially the second mesiobuccal canal (MB2) in first molars) having the highest frequency. AP was found in 81.0% of cases with missed canals compared to 45.5% of cases with all canals treated (p < 0.001), indicating a strong correlation between disease and missed canal presence. Another significant factor was the quality of the coronal restoration; restorations with open margins, recurrent caries, or loss of integrity were linked to a significantly higher prevalence of AP (72.3%) than restorations with adequate sealing (38.5%, p < 0.001). These associations are summarised in Table 3 (see PDF). Once age, sex, and tooth location were taken into account, multivariable logistic regression validated these associations. While patient-related variables were not statistically significant, AP was significantly predicted by inadequate obturation length (aOR = 2.47; 95% CI: 1.36-4.47), poor obturation density (aOR = 2.11; 95% CI: 1.15-3.88), missed canals (aOR = 3.56; 95% CI: 1.54-8.21), and poor coronal restorations (aOR = 2.98; 95% CI: 1.63-5.47). Excellent reproducibility was shown by reliability analysis. While intra-examiner reliability for volumetric lesion measurement achieved intraclass correlation coefficients greater than 0.90, inter-examiner agreement was high for both AP detection (kappa = 0.83) and obturation quality assessment (Cohen's kappa = 0.87). When combined, these results demonstrate that the periapical condition of molars is significantly influenced by the technical quality of coronal restoration and root canal therapy. The main causes of persistent apical periodontitis are still missed canals and suboptimal obturation, which emphasises the significance of careful canal identification, superior obturation, and long-lasting coronal sealing for the best long-term endodontic results.

## Discussion:

The association between the quality of endodontic treatment and the incidence of apical periodontitis (AP) has been consistently highlighted across several CBCT-based investigations. Meirinhos *et al.* [12] reported that inadequate root canal filling length and poor coronal restoration significantly increase the risk of AP, underscoring the importance of precise obturation and restorative techniques. Similarly, Do Carmo *et al.* [13] and Carrion *et al.* [14] observed a high prevalence of missed canals in posterior teeth, particularly in the mesiobuccal roots of maxillary molars, which show a strong association with periapical pathology. Further supporting these findings, Aysal *et al.* [15] and Çikman *et al.* [16] emphasised the diagnostic superiority of CBCT, confirming its enhanced accuracy in assessing both the technical quality of root canal therapy and the presence of AP. Alnowailaty and Alghamdi [17], along with Alotaibi *et al.* [18], reinforced that untreated or missed canals remain major contributors to periapical disease, with maxillary molars-especially their mesiobuccal roots-being particularly susceptible. Rouhani *et al.* [19] also demonstrated the high frequency of missed canals across various tooth types, supporting the essential role of CBCT in their detection. Lewusz-Butkiewicz *et al.* [20] validated the usefulness of CBCT in epidemiological assessment, offering valuable insights into periapical health trends and treatment outcomes. Population-specific studies, such as that by Alhailaa *et al.* [21], further revealed that the prevalence of AP increases markedly in cases with poor-quality endodontic treatments-findings consistent with global trends. Moreover, Pietrzycka *et al.* [22] highlighted the influence of operator experience on obturation quality and lesion incidence, indicating the critical need for adequate training and skill development. Finally, Sahin and Gündüz [23] concluded that suboptimal treatment quality significantly worsens AP in molars, reaffirming the need for strict adherence to technical standards and meticulous treatment planning in endodontics.

## Conclusion:

The prevalence of apical periodontitis is directly impacted by the calibre of endodontic treatment. Apical pathology risk is considerably decreased by adequate obturation and appropriate coronal sealing. Persistent disease is still significantly predicted by missed canals and subpar treatment.
